# Differences in reward processing between putative cell types in primate prefrontal cortex

**DOI:** 10.1371/journal.pone.0189771

**Published:** 2017-12-19

**Authors:** Hongwei Fan, Xiaochuan Pan, Rubin Wang, Masamichi Sakagami

**Affiliations:** 1 Brain Science Institute, Tamagawa University, Machida, Tokyo, Japan; 2 Oracle (China) Software Systems Co., Ltd., Chaoyang District, Beijing, P.R. China; 3 Institute for Cognitive Neurodynamics, East China University of Science and Technology, Shanghai, P.R. China; University of Toyama, JAPAN

## Abstract

Single-unit studies in monkeys have demonstrated that neurons in the prefrontal cortex predict the reward type, reward amount or reward availability associated with a stimulus. To examine contributions of pyramidal cells and interneurons in reward processing, single-unit activity was extracellularly recorded in prefrontal cortices of four monkeys performing a reward prediction task. Based on their shapes of spike waveforms, prefrontal neurons were classified into broad-spike and narrow-spike units that represented putative pyramidal cells and interneurons, respectively. We mainly observed that narrow-spike neurons showed higher firing rates but less bursty discharges than did broad-spike neurons. Both narrow-spike and broad-spike cells selectively responded to the stimulus, reward and their interaction, and the proportions of each type of selective neurons were similar between the two cell classes. Moreover, the two types of cells displayed equal reliability of reward or stimulus discrimination. Furthermore, we found that broad-spike and narrow-spike cells showed distinct mechanisms for encoding reward or stimulus information. Broad-spike neurons raised their firing rate relative to the baseline rate to represent the preferred reward or stimulus information, whereas narrow-spike neurons inhibited their firing rate lower than the baseline rate to encode the non-preferred reward or stimulus information. Our results suggest that narrow-spike and broad-spike cells were equally involved in reward and stimulus processing in the prefrontal cortex. They utilized a binary strategy to complementarily represent reward or stimulus information, which was consistent with the task structure in which the monkeys were required to remember two reward conditions and two visual stimuli.

## Introduction

Cortical neurons are usually classified into two main types: pyramidal cells and interneurons. These two types of neurons may differ in morphology, neurotransmitter types, electrophysiological properties, and so on [1–3]. It has been reported that pyramidal cells had broader spike waveforms than did interneurons in intracellular recordings [2,4]. Based on this evidence, neurons with broad spike waveforms are classified as putative pyramidal cells and neurons with narrow spike waveforms as putative interneurons in extracellular recording experiments to investigate their functions in behavioral tasks [5–8]. It has been reported that putative pyramidal cells and interneurons in the prefrontal cortex (PFC) may have distinct functional roles in higher-order cognitive functions, such as working memory [9] and category representation [10], sensory information representation and decision-making [11–13]. Several studies have demonstrated that the interaction between putative pyramidal cells and interneurons played a critical role in shaping spatial working memory [14] and the temporal information flow in the PFC [15]. In a numerical categorization task [10], both putative pyramidal cells and interneurons in the PFC showed numerical turning properties. Additionally, feed-forward inhibitory inputs from putative interneurons to putative pyramidal cells sharpened the tuning curves of pyramidal cells. These results suggest that microcircuits between pyramidal cells and interneurons subserve cognitive operations in the PFC [13,16].

It is known that prefrontal neurons are involved in reward processing [17–19]. Many single-unit studies in monkeys have demonstrated that activities of prefrontal neurons predicted or expected the reward type [20], the reward amount [21,22] or the reward availability [23] that was associated an external stimulus. However, it remains elusive whether and how putative pyramidal cells and interneurons in the PFC encode reward information. In this study, we focused on two questions: 1) whether both putative pyramidal cells and interneurons encode reward information in the PFC. 2) If the two classes of neurons can do so, what type of reward information do they represent, respectively? To answer the first question, neuronal activities on trials containing different reward conditions are compared to identify whether a neuron encodes reward information or not. If the neuron has activity changes in two reward conditions (e.g., large vs. small rewards), we could say this neuron represents reward information. But we don’t know what type of reward information (e.g., large, small or both) this neuron encodes. To answer the second question, neuronal activity should be compared between pre- and post-reward cue periods in the same trials. For example, a reward neuron only encodes the large reward information if it shows activity changes before and after the cue onset in large reward trials, but not in small reward trials.

To investigate these questions, we recorded single-unit activity extracellularly in the lateral PFCs (LPFCs) of four monkeys while they performed a reward prediction task [24,25], in which two classes of visual stimuli were associated with two different amounts of reward. The recorded neurons were classified into putative pyramidal cells and interneurons based on their shapes of action potential waveforms. It was found that both putative pyramidal neurons and interneurons encoded reward and stimulus information, but that they might use different mechanisms to do so. The putative pyramidal cells with low baseline firing rates increased their discharge rates to encode the preferred reward and stimulus information; whereas the putative interneurons with high baseline firing rates decreased their activities to represent the non-preferred reward and stimulus information. In addition, both the putative pyramidal cells and interneurons showed similar reliability in discrimination between the preferred and non-preferred reward and stimulus information. Our findings suggest that the two classes of PFC neurons had important but distinct functional roles in the PFC neuronal circuits involved in reward processing.

## Materials and methods

### Subjects

Four male Japanese monkeys (Macaca fuscata) served as subjects in this study (Hop, 7.5kg, Tap, 6.5kg, Tom, 8.9kg and Zep, 8.5kg). A head-holder and two recording chambers (one in each hemisphere) were implanted for each monkey under aseptic techniques with ketamine (4.6–6.0 mg/kg by intramuscular injection) and sodium pentobarbital (Nembutal, 4.5–6.0 mg/kg by intravenous injection) anesthesia. The size of each chamber was 40 mm (length, anterior-posterior) × 30 mm (width, lateral-medial), and these were implanted with their centers located at around the end of the principal sulcus.

During each experimental session, the monkey was seated in a primate chair (with its head fixed) inside a completely enclosed sound-attenuated and electrically shielded room. A 21-inch CRT display (FE220, NEC, Japan) with 60Hz refresh rate was set at a distance of 60.0 cm in front of the monkey for the presentation of visual stimuli. Eye movements were monitored by the Eyelink2 system (SR Research Ltd, Mississauga, Canada) with 500 Hz sample rate. All stimulus presentation and behavioral procedures were controlled by the TEMPO system (Reflective Computing, USA).

### Ethics statement

All experimental protocols in this macaque study were approved by the Animal Care and Use Committee (H26-42) at Tamagawa University and were in accordance with the National Institutes of Health’s Guide for the Care and Use of Laboratory Animals. The experimental period of this study was 6 years, from 2005 to 2011. The four monkeys (Hop, Tap, Tom and Zep) were recorded. The two monkeys (Hop and Zep) were bought from HAMRI Co. Ltd (http://www.hamri.co.jp/index.html) and the other two were bought from The National Bioresource Project (NBRP) (https://nihonzaru.jp/aboutus_2_e.html). The monkeys were bred and housed in their colonies before being moved to the laboratory. After the monkeys being moved to the laboratory, they were housed in individual primate cages for another one year prior to this study. The primate cages were arranged into two lines along the two sides of walls in an air-conditioned room. Food and water were available ad libitum from the cage. Each cage was equipped with a platform along the middle of the wall and was large enough for the monkeys to move up and down freely. Each monkey could see others’ faces in the opposite cages. The body weight of the monkeys was measured and vegetables and fruits were provided daily. Additional water, vegetables and fruits were delivered during weekends as reward after experiments. During training and recording sessions, water was delivered as reward for the monkeys in their correctly performed trials. Each monkey could receive about 400ml water in each experimental session. A head holder was first implanted for each monkey in order to train the monkey to learn the task. It cost about 6 months. After the completion of training, the chambers were implanted for recording. The time frame from the implantation of the recorded chambers to the completion of this experiment is shown below for each monkey: Hop: from July 18, 2005 to January 27, 2007; Zep: from December 07, 2005 to January 17, 2007; Tap: from February 07, 2007 to July 08, 2008; Tom: from December 14, 2009 to February 15, 2011. The inside of the recording chambers were regularly washed with saline and antibiotic ointment was put on the dura matter to keep the inside clean. The monkeys were deeply anesthetized with an overdose of sodium pentobarbital (40 mg/kg, intravenous injection) when being sacrificed.

### Behavioral task

A detailed description of the behavioral task can be found in Pan et al. [24,25]. Here we briefly introduce the sequential paired-association task with asymmetric reward schedules.

#### Sequential paired-association task

**Fig 1:**
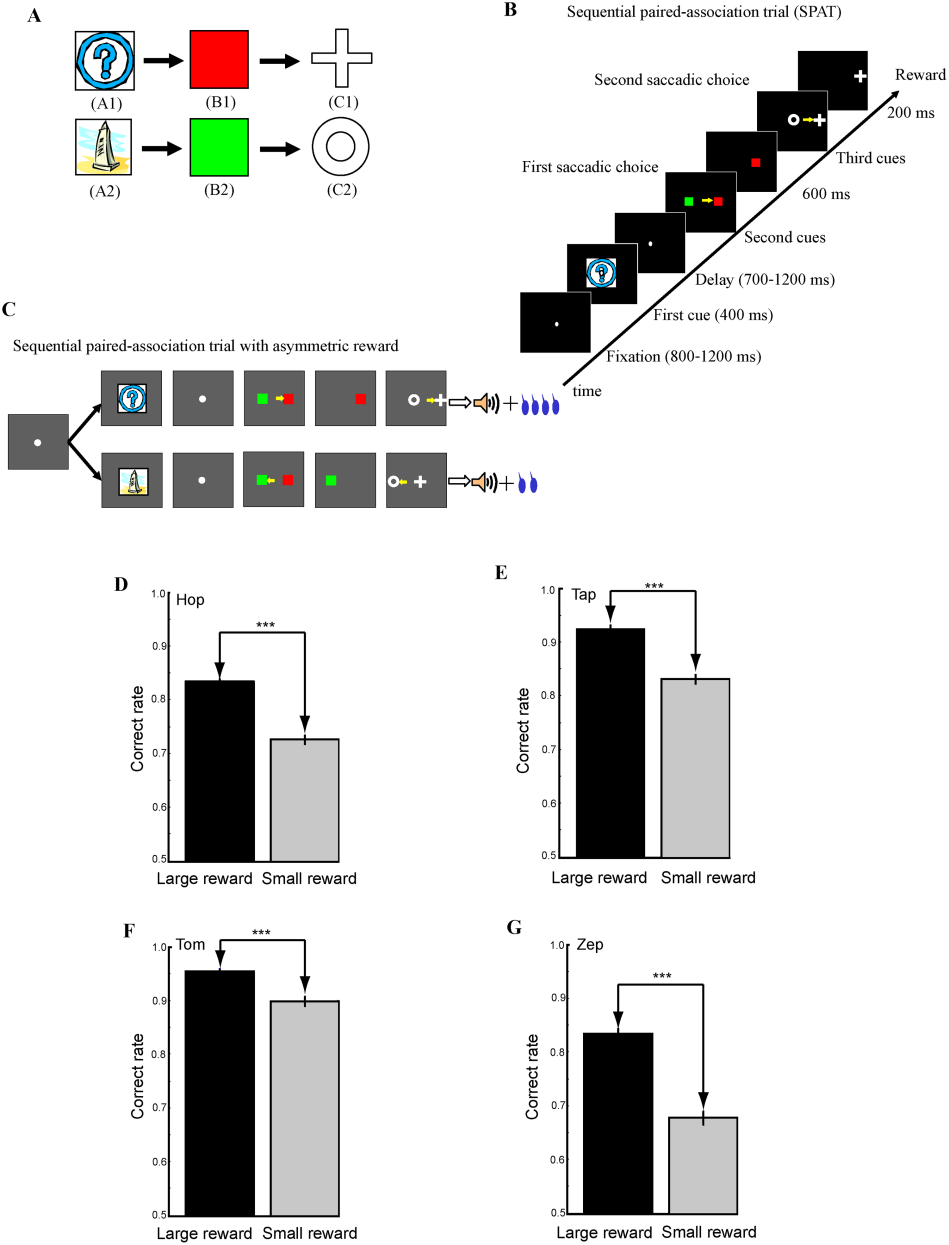
The sequential paired-association task and behavioral performance of four monkeys. (A) Two stimulus-stimulus associative sequences (A1→B1→C1 and A2→B2→C2) learned by the monkeys. (B) The schematic illustrates the time course of a single SPAT. The animals had to make two choices (First choice: selection B based on A and second choice: selection C based on B) by saccadic eye movements (as denoted by small yellow arrows) in a trial. (C) An asymmetric reward schedule was used in a block. In this given block, a correct performance of A1-sequence was paired with a large reward (0.4ml), while a correct performance of A2-sequence was paired with a small reward (0.2ml). The sequence-reward contingency was pseudo-randomized between blocks. (D)-(G) Average behavioral performance (% correct) across recording sessions for the four monkeys, Hop (D), Tap (E), Tom (F) and Zep (G). The correct rates (correct trials divided by total trials) of the first saccadic choice in SPATs were calculated. The black bars indicate correct rates in the trials with a large amount of reward, while the grey bars represent the correct rates in the trials with a small amount of reward. *** *P* < 0.001 (Mann Whitney *U* test). Error bars: SEM.

The four monkeys were trained to learn two associative sequences (Fig 1A) in a sequential paired-association task (Fig 1B). The two correct sequential associations were: A1→B1→C1 and A2→B2→C2. The onset of a fixation spot (0.21° of visual angle) at the center of the monitor indicated the start of a sequential paired-association trial (SPAT, Fig 1B). The monkey had to fixate on the fixation spot for a random duration (800–1200ms). Subsequently the first stimulus cue (e.g. A1) was presented at the center of the display for 400ms. Then a delay period followed (700–1200ms). After the delay period, the fixation spot disappeared, and at the same time the second set of cues (B1 and B2) were presented pseudo-randomly at left and right positions on the CRT (6° of visual angle from center). The monkey then made a saccade to the target cue (e.g. B1); this was denoted as the first choice. Immediately after the correct first choice, the distracter (e.g. B2) was removed from the display, and the monkey continued fixating on the target cue (B1) for another 600ms. After the disappearance of the target cue (B1) the third set of cues (C1 and C2) were simultaneously displayed pseudo-randomly to the left and right of where the target cue (B1) had appeared (5° of visual angle from the central position of B1). This instructed the monkey to make a further saccadic eye movement to the correct target cue (e.g. C1) in the second choice. After two correct choices, the monkey received a drop of water as reward and an auditory tone of 1kHZ at the end of the trial.

The saccade was judged to be correct if the eye position stayed for at least 200ms inside a virtual window (3° × 3° of visual angle) centered on the position of the target stimulus. The monkey had to keep its fixation inside the virtual fixation window during the fixation, cue presentation, and delay periods. If it moved its fixation out of this window the trial was rejected as a fixation break. When the monkey made a fixation break or an erroneous choice, the trial was aborted, and a high tone of 4k Hz indicated an error. In these cases, after a longer inter-trial interval (ITI) (6s, the normal ITI after a correct trial was 3s), the same trial was repeated until the monkey was able to complete it correctly. The repeated trials after an error were referred to as correction trials. Through SPAT training the four monkeys learned the two associative sequences correctly: A1→B1→C1 and A2→B2→C2.

#### Asymmetric reward schedules

After completing learning of the two associative sequences, the monkey was required to perform the sequential paired-association task with asymmetric reward schedules block by block (Fig 1C). In a given block, one associative sequence (e.g. A1→B1→C1) was paired with a large amount of reward (0.4ml) and the other sequence (e.g. A2→B2→C2) was paired with a small amount of reward (0.2ml). The monkey would receive the large reward if it performed the sequence of A1→B1→C1 correctly in this example block. It would receive the small reward even if it performed the sequence of A2→B2→C2 correctly. In another example block, the sequence-reward contingency might be reversed. Now the sequence of A1→B1→C1 would be paired with the small reward and the sequence of A2→B2→C2 with the large reward. At the beginning of each block, reward instruction trials (in which C1 and C2 were paired with different amounts of reward) were introduced to indicate which sequence would have the large reward and which one would have the small reward. Therefore, the monkeys knew the sequence-reward contingency from the first SPAT in each block [24,25].

### Data recording

Action potentials of single neurons were recorded extracellularly with tungsten electrodes (FHC, Bowdoinham, ME, 0.8–1.5MΩ) from the LPFCs of the four monkeys. The microelectrode was inserted via a guide tube through a grid system (holes: 0.6 mm wide and 1.0 mm apart from center to center; Nakazawa, Tokyo, Japan) into the cortical surface; then the electrode was advanced into the cortex by means of the NAN-electrode-drive (NAN-instruments LTD, Israel). The Plexon MAP system (Plexon Inc, Texas, USA) was used to amplify action potentials and discriminate individual spike waveforms online, and then saved the spike timing and selected waveforms (in a 1200μs-window for Tom, and an 800μs-window for the other three monkeys) on the Plexon PC together with task events.

### Data analysis

Custom-made Matlab programs were used to carry out off-line analysis on a PC. We calculated the behavioral performance of the first choice in SPATs (correct rate: correct trials divided by total trials) in each recording session, then averaged the correct rates across sessions. Because we used the correction method in this experiment, we excluded the data in correction trials (the repeat trials presented after each error) when calculating the correct rate. For the analysis of neuronal data, we used only the activity in correct trials, and excluded the data in erroneous and correction trials.

#### Selection and classification of neurons

Neurons were selected for analysis based on the following criteria in this study: (1) each neuron was clearly isolated from the other neurons and from multiunit activity based on clear clustering in the principle components of waveforms (Offline Sorter, Plexon Inc.). (2) Each unit showed a clear spike refractory period. (3) The waveforms of each neuron exhibited a downward voltage deflecting (a trough, negative related to baseline) followed by an upward voltage deflecting with a clear peak. (4) LPFC neurons with deviated waveforms or saturated amplitude waveforms were excluded from analysis.

A function of k-means classifier (k = 2, squared Euclidean distance) in Matlab was used to classify neurons into two groups based on the shape of averaged spike waveforms. In order to reduce possible effects of different amplitude and timings of minimum of waveforms, we normalized the waveforms of each neuron by the differences between their peak and trough values and aligned them by their minimum. The k-means algorithm (k = 2) aims to partition the spike waveforms into 2 clusters in which each waveform belongs to the cluster with the nearest mean waveform, so as to minimize the variance of waveforms within the cluster [26]. The k-means algorithm does not examine any statistical significance among k clusters or does not necessarily find global optimal clusters. Therefore we calculated waveform durations (between the trough and peak in the averaged waveform) to further confirm that the classification by k-means (k = 2) was appropriate. The advantage of k-means (k = 2) classifier is that this algorithm objectively classifies spike waveforms into two clusters on the basis of their shapes, without the requirement of a pre-determined border between the two clusters. We referred to the neurons that had the smaller mean spike width as narrow-spike (NS) neurons, and to the neurons that had the larger mean spike width as broad-spike (BS) neurons.

#### Visually evoked response latency

To determine the visually evoked response latency for each neuron, its averaged spike density histogram was derived with 1ms resolution, and smoothed by a Gaussian envelope with σ = 15ms for all trials. We calculated the mean and standard deviation (SD) of firing rates during a 200ms-time-window prior to the first cue onset across all trials for each neuron, and set the threshold at the mean of baseline firing rates plus 3 SDs. If the neuronal activity exceeded the threshold for three consecutive time bins after the first cue onset, the response latency was defined as the duration from the first cue onset to the first of these time bins. If a neuron was unable to reach this criterion, it was excluded from the latency count.

#### Inter-spike interval (ISI) distribution

We used the FieldTrip open source Matlab toolbox [27] to calculate the ISI distribution and local variation for each neuron. The ISI distribution of each neuron was fitted by a Gamma distribution and its parameters such as the peak mode were estimated.

#### Classification of neurons by two-way analysis of variance (ANOVA)

To characterize response properties of each neuron, a two-way ANOVA was used to analyze its activity with the two factors of stimulus (A1 vs. A2) and reward (large vs. small) in two time epochs prior to the second cues: the cue and delay periods, respectively. The cue period was from 100ms to 500ms after the first cue onset, 100ms shifted from the cue onset because some neurons responded to the cue offset. The delay period was from 500ms to 900ms after the first cue onset. There was no gap between the cue and delay periods. Depending on results of the ANOVA (P < 0.01), neurons were classified into three types: stimulus neurons (S-neurons), reward neurons (R-neurons) and stimulus-reward neurons (SR-neurons) [25]. S-neurons showed a significant main effect of stimulus (P < 0.01), but neither a main effect of reward nor interaction between the two factors. Neurons which only showed a significant main effect of reward (P < 0.01) were classified as R type. This type of neurons predicted the amount of reward associated with a stimulus regardless of stimulus properties. SR-neurons showed a significant interaction between stimulus and reward and/or significant main effects of both stimulus and reward. The activity of each neuron was analyzed in the cue and delay periods, respectively. A neuron could be assigned as the same type of neuron (R-, S-, or SR-neurons) repeatedly in the two periods. For each R-neuron, we referred to the reward condition (large or small reward) that elicited the higher firing rate by the first cue stimuli (A1 and A2) as the preferred reward condition, and the other as the non-preferred reward condition. Similarly, for each S-neuron, its preferred stimulus was defined as the stimulus that could evoke higher activity than the other stimulus (the non-preferred stimulus).

The method of two-way ANOVA described above compared the neural activity on different trials (i.e., in different reward conditions) to determine whether the neuron was an R-neuron or not. Once a neuron was identified as an R-neuron, we further examined what type of reward information (the preferred reward or the non-preferred reward) that neuron encoded. To answer this question, we compared the baseline activity in the fixation period (-300-0ms prior to the first cue onset) with the activity in the cue period or in the delay period. The baseline activity had no reward information about the current trial. If one reward neuron showed activity changes between the fixation period and the cue period or the delay period in the preferred reward condition, it encoded the preferred reward information. If this neuron showed no activity changes between the pre- and post-stimulus periods in the non-preferred reward condition, it should not represent the non-preferred reward information. If a neuron showed significant activity differences between the two periods in both the reward conditions, it could encode the preferred and non-preferred reward information simultaneously. From this sense, R-neurons can represent the preferred reward information or the non-preferred reward information or both of them. We observed both the BS and NS R-neurons showed visually evoked response to the presentation of the first cue in the two reward conditions. This visually evoked response would take strong effect on comparison between the baseline activity and the cue period activity. In the current task design, we were unable to separate the evoked response from the reward-related signal. To avoid this effect, the cue period was divided into two time epochs: the early cue period (100–300ms from the first cue onset) and the late cue period (300–500ms from the first cue onset). There might be more visually evoked activities in the early cue period, and less in the late cue period. We compared the activity in the fixation period with the activity in the late cue period to demonstrate the reward information represented in each R-neurons. The activity in the delay period should not include the visually evoked response, and it was compared directly with the baseline activity.

#### Population peristimulus histograms

To generate the population histogram we first calculated the firing rate in every 1ms bin within each trial, then averaged the firing rate in every bin across all trials for each neuron and across population neurons, and finally smoothed the data using a Gaussian envelope with σ = 30ms.

#### Reward discrimination reliability and latency

To quantify reward discrimination ability of each R-neuron, we performed a receiver operating characteristic (ROC) analysis [28] by comparing activity on trials containing the two reward conditions (preferred vs. non-preferred) in the cue period or in the delay period. The ROC analysis measures the degree of overlap for two distributions of neuronal activity. The advantage of this method is that it is independent of the baseline firing rate and dynamic ranges; it also does not require a normal distribution assumption. In our database, the activity of R-neuron in the preferred reward condition was larger than the activity in the non-preferred reward condition. This ROC analysis calculated AROC (area below the ROC curve) values ranging between 0.5 and 1. AROC = 0.5 indicated activity that did not differ between the preferred and non-preferred reward conditions, while AROC > 0.5 indicated higher activity in the preferred reward condition. A larger AROC value indicated better reliability to discriminate the two reward conditions.

To examine the reward discrimination latency of each R-neuron, a sliding ROC analysis was performed in a 100ms window stepped with every 10ms interval. The window started from the fixation period of the trial (600ms prior to the first cue), and stepped to the end of the delay period. Significance of AROC values in each time window was evaluated by bootstrap test [29], shuffling large and small reward trial labels. This process was repeated 1000 times to generate a distribution of shuffled AROC values. The original AROC value was significant if it fell within the top or bottom 2.5% of the distribution (P < 0.05). The reward-discrimination latency was defined as the duration from the first cue onset to the center of the first significant window of at least seven consecutive significant windows. If a neuron was not able to reach this criterion, it was excluded from the latency count.

The same method was applied to calculate AROC values and discrimination latencies for the S-neurons and SR-neurons, respectively. For each S-neuron, the two spike rate distributions for the preferred and non-preferred stimuli were compared. For each SR-neuron, AROC values for reward and stimulus were examined separately. Trials only with the preferred stimulus were selected to calculate AROC values of reward because SR-neurons did not discriminate the two reward conditions on trials with the non-preferred stimulus. And trials only with the preferred reward were chosen to calculate AROC values of stimulus because they did not distinguish the preferred from non-preferred stimuli in the non-preferred reward trials.

#### Multiple linear regression analysis of reward-related activity

To examine the correlation between neuronal activity with stimulus, reward size and saccadic parameters, we performed a multiple regression analysis for each reward-related neuron with two models in the cue and delay periods, respectively:

$$Y=a_{0}+a_{1}Stim\_type+a_{2}\text{Re}ward\_type$$ (1)

$$Y=a_{0}+a_{1}left\_RT+a_{2}right\_RT+a_{3}left\_peak\_V+a_{4}right\_peak\_V$$ (2)

where Y is the firing rate of a neuron in the cue period or in the delay period, left_peak_V is the peak saccadic velocity to the left direction in the first choice, right_peak_V is the peak velocity to the right direction, left_RT is saccadic response time to the left direction, right_RT is response time to the right direction, a_0_ is a constant in the models, and a_1_, a_2_, a_3_, a_4_ are coefficients. The variable Stim_type indicated the first cue stimulus (A1 or A2), and its value was set 1 or 0 for preferred or non-preferred stimulus, respectively. The variable Reward_type was set 1 or 0 for trials with large or small rewards. If neuronal activity in the cue period or in the delay period represents reward type and stimulus information, but not motor parameters, then Model 1 would fit well with the data. If activity just represents saccadic parameters, then Model 2 would fit well with the data.

## Results

The activity of single LPFC neurons was recorded extracellularly in the four monkeys while they performed the sequential paired-association task with asymmetric reward schedules block by block (Fig 1C). The behavior of each monkey was systematically influenced by the amount of reward. The correct rate of the first choice (selection of B_1_ or B_2_ on the basis of the first cue A_1_ or A_2_) was calculated in the large and small reward trials separately. The four monkeys consistently showed significantly higher correct rates in large than small reward trials (Fig 1D–1G, Mann Whitney *U* test, *P* < 0.001), indicating the monkeys correctly predicted reward values associated with the first cue stimuli (A1 and A2).

### Identification of broad-spike and narrow-spike neurons

**Fig 2:**
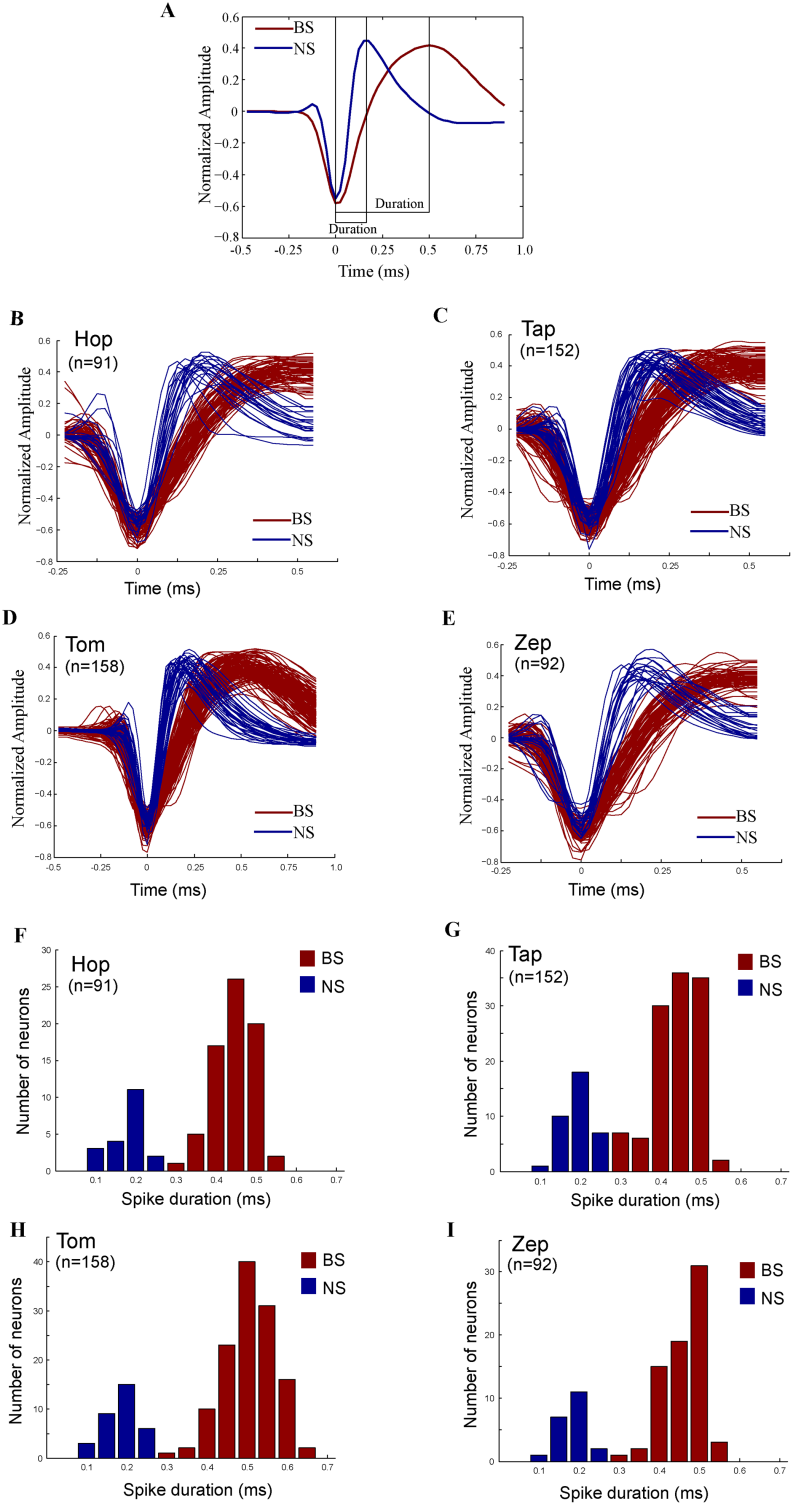
Identification of BS and NS neurons. (A) Average spike waveforms of one BS neuron (red curve) and one NS neuron (blue curve) recorded in an electrode simultaneously. The duration is calculated between the trough and peak of the average waveform. (B)-(E) Average spike waveforms of two groups of neurons classified by k-means (k = 2) classifier for the four monkeys, Hop (B), Tap (C), Tom (D) and Zep (E), respectively. The red waveforms indicate BS neurons and the blue ones represent NS neurons. (F)-(I) Distributions of durations for the four monkeys, Hop (F), Tap (G), Tom (H) and Zep (I). The red bars indicate durations of BS neurons and the blue bars indicate durations of NS neurons. The distributions were not unimodal (Hartigan’s dip statistic test, P < 0.01).

A total of 493 neurons were selected for further analysis based on the four criteria (see Materials and methods, 91 from Hop, 152 from Tap, 158 from Tom and 92 from Zep). To classify neurons based on their waveforms, the action potential waveforms of each neuron were normalized by the differences between their peak and trough values, aligned by their troughs, and then averaged. Across our sampled neurons, waveforms had similar biphasic shapes but varied in duration, defined here as the time between the trough and peak of the averaged waveform (Fig 2A).

All neurons were objectively classified into two groups by a linear classifier (k-means, k = 2) based on their shapes of avegared waveforms (see Materials and methods). Waveforms of the two classified neurons are separately presented in Fig 2B–2E for each monkey. We found that there were 383 (77.7%) BS neurons and 110 (22.3%) NS neurons in the database.

The duration between the trough and peak in the averaged waveform was calculated for each of the neurons. Distributions of the durations were bimodal (Fig 2F–2I, Hartigan’s dip statistic test, P < 0.01 in each of the four monkeys). Durations of NS neurons (blue bars) were significantly shorter than durations of BS neurons (red bars) in each of the monkeys, respectively (for Hop, the median duration of NS neurons: 0.2ms, the median duration of BS neurons: 0.475ms; for Tap, 0.2ms, 0.45ms; for Tom, 0.2ms, 0.525ms; for Zep, 0.2ms, 0.475ms. Mann Whitney U test, P < 10^−6^).

**Fig 3:**
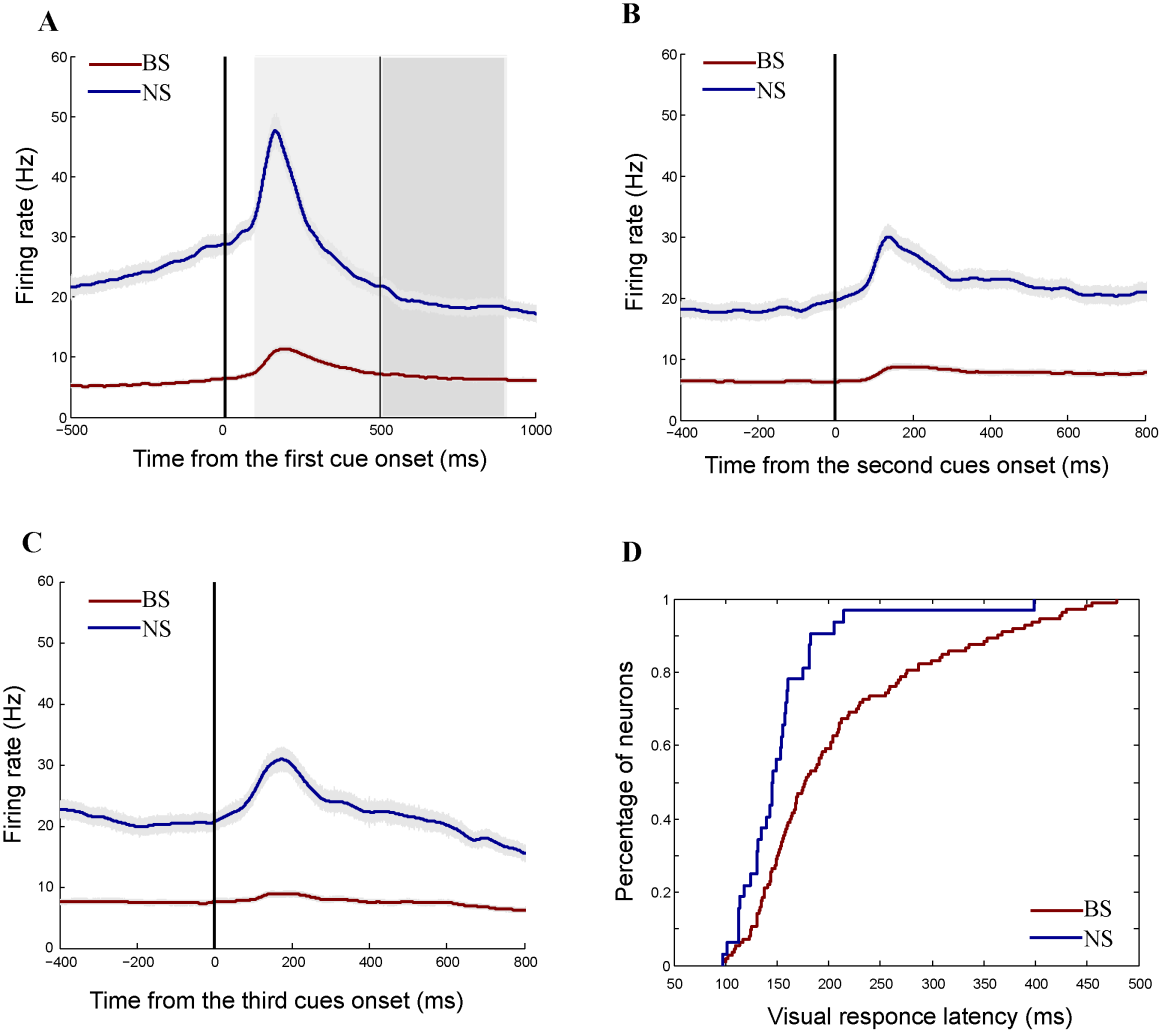
Characteristic responses of BS and NS neurons. (A)-(C) Population histograms of BS (red curves) and NS (blue curves) neurons aligned at the first cue onset (A), the second set of cues onset (B) and the third set of cues onset (C). The shaded areas around the curves indicate SEM. The two gray areas in (A) indicate the cue and delay periods, respectively. The NS neurons show significantly higher firing rates in all task periods than did the BS neurons (Mann Whiney *U* test, P < 0.001). (D) Cumulative curves of visual response latencies for BS neurons (red curve) and NS neurons (blue curve). NS cells showed faster visual responses than did BS neurons (Mann Whitney *U* test, *P* < 0.001).

Several different physiological properties were found between the BS and NS neurons. First, NS cells showed significantly higher discharges than did BS cell in all task periods (Fig 3A–3C, Mann Whiney *U* test, P < 0.001). Second, NS cells showed faster visual responses than did BS neurons (Fig 3D, the median latencies: 177.0 ms, 145.5 ms for BS and NS neurons, respectively, Mann Whitney *U* test, *P* < 0.001). Third, we did not observe burst firing patterns in either the population of BS cells or in the population of NS cells to be characterized by local variation (LV) values [30] that were less than one (the median LV values: 0.813, 0.534 for BS and NS neurons). But the LV values significantly differed between cell types (Mann Whitney *U* test, P < 0.001). Fourth, in ISI distributions, peak mode times of BS cells were significantly longer than that of NS cells (the median model time: 32ms, 15ms for BS and NS cells, Mann Whitney *U* test, P < 0.001). Overall, the observed differences in spike waveforms, durations, response properties and spike train statistics between the BS and NS cells were consistent with the distinct characteristics of pyramidal cells and interneurons reported in previous studies [31,32], suggesting that most of the BS neurons were pyramidal cells and most of the NS cells were interneurons in our database [33–35].

### Classifying BS and NS neurons into S-, R- and SR-neurons

To examine whether the BS and NS neurons were selective response to reward and stimulus properties, we performed a two-way ANOVA (two factors: stimulus (A1 vs. A2) x reward condition (large vs. small)) to analyze the activity in the cue (100-500ms from the first cue onset) and delay periods (500-900ms from the first cue onset), respectively. Based on results of the two-way ANOVA (P < 0.01), both the BS and NS neurons were classified into R-, S- and SR-neurons in the cue and delay periods (see Materials and methods).

In the cue period, there were 59 R-neurons, 35 S-neurons, and 37 SR-neurons out of 383 BS neurons (Table 1). Within 110 NS neurons, 18 of them were R-neurons, 13 were S-neurons, and 24 were SR-neurons (Table 2). We calculated the incidence of each type of neurons in the BS and NS classes. Either the incidences of R-neurons or the incidences of S-neurons did not differ significantly between the two classes (BS R-neuron: 15.4% (59/383), NS R-neuron: 16.4% (18/110), *P* = 0.8071; BS S-neuron: 9.1% (35/383), NS S-neuron: 11.8% (13/110), *P* = 0.4034, χ^2^-test). However, we found a significantly higher proportion of SR neurons in the NS class than in the BS class (BS SR-neuron: 9.7% (37/383), NS SR-neuron: 21.8% (24/110), χ^2^ -test, *P* < 0.001).

**Table 1:** Classification of LPFC broad-spike (BS) neurons in the four monkeys.

Monkey	BS neurons	BS R-neurons		BS S-neurons		BS SR- neuron	
		Cue period	Delay period	Cue period	Delay period	Cue period	Delay period
Hop	71	9	9	7	1	5	3
Tap	116	18	21	14	8	12	8
Tom	125	24	28	5	3	13	7
Zep	71	8	14	9	5	7	6
**Total**	383	59	72	35	17	37	24

**Table 2:** Classification of LPFC narrow-spike (NS) neurons in the four monkeys.

Monkey	NS neurons	NS R-neurons		NS S-neurons		NS SR-neuron	
		Cue period	Delay period	Cue period	Delay period	Cue period	Delay period
Hop	20	3	4	4	0	4	1
Tap	36	3	10	3	1	11	3
Tom	33	9	9	2	1	3	2
Zep	21	3	7	4	0	6	0
**Total**	110	18	30	15	2	24	6

In the delay period, we found the 383 BS neurons to include 72 (29) R-neurons, 17 (5) S-neurons, and 24 (10) SR-neurons (Table 1). The number in the parentheses indicated the number of neurons that were also identified as the same type in the cue period. The 110 NS neurons included 30 (11) R-neurons, 2 (0) S-neurons, and 6 (4) SR-neurons (Table 2). None of the incidences of R-neurons, S-neurons or SR-neurons in the BS class singnificantly differed from that in the NS class (χ^2^-test, *P* > 0.05). These results indicated that the NS neurons, as well as the BS neurons, played functionals roles in encoding information of reward, stimulus and their interaction in the LPFC. We further analyzed response properties of R-, S- and SR-neurons in the two cell classes.

### BS and NS R-neurons

#### Population histograms of BS and NS R-neurons

**Fig 4:**
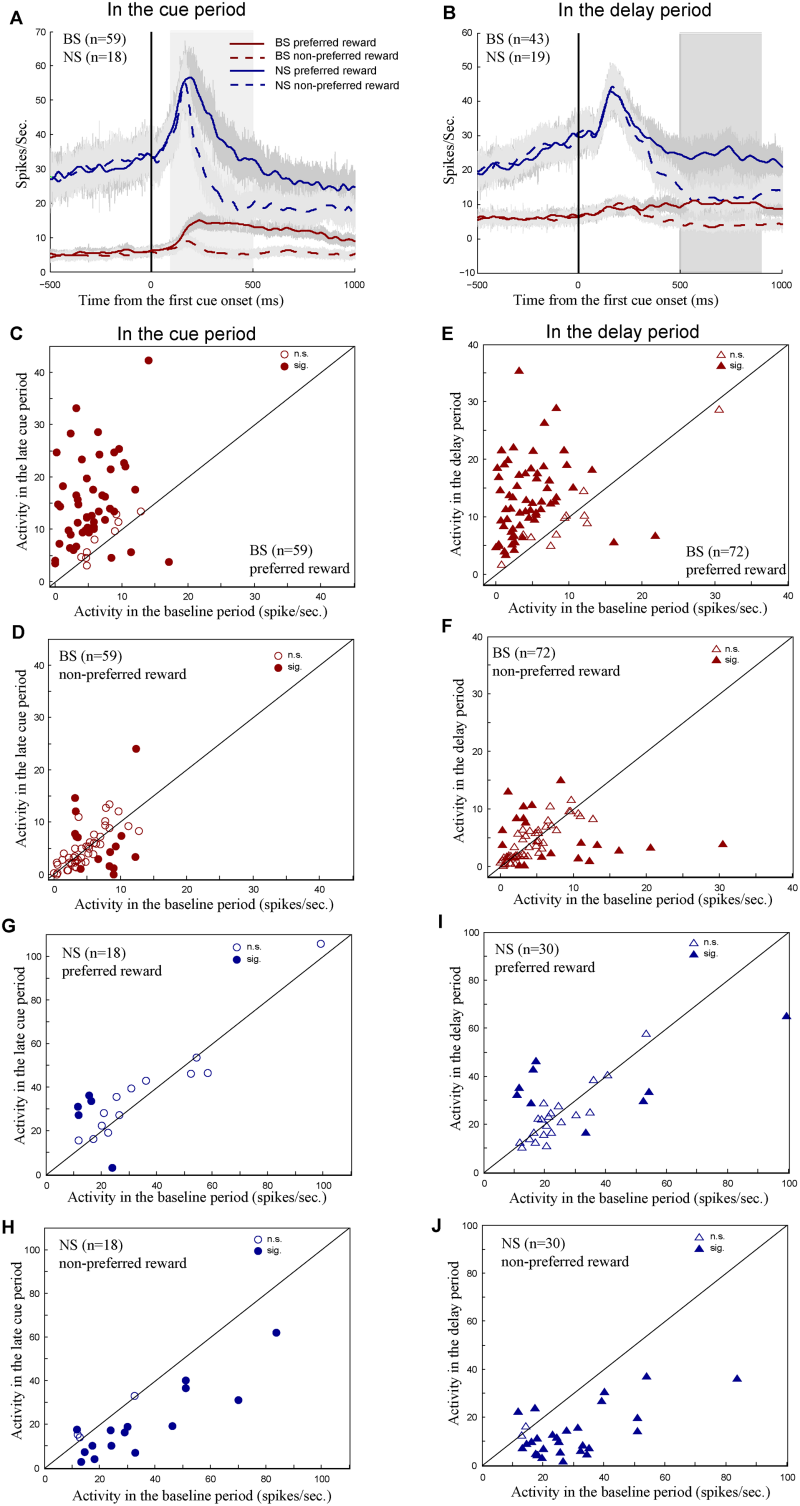
Response properties of BS and NS R-neurons in the cue and delay periods. (A) Population histograms of BS (red curves) and NS (blue curves) R-neurons identified in the cue period. (B) Population histograms of BS and NS R-neurons identified only in the delay period not in the cue period. The activity of each neuron was sorted by the two reward conditions: the preferred reward condition (solid curves) and the non-preferred reward condition (dashed curves). The shaded areas around the curves indicate SEM. The gray area in (A) is the cue period, and the gray area in (B) is the delay period. (C)-(D) Scatterplots of the baseline activity of BS R-neurons against the late cue activity in the preferred (C) and non-preferred (D) reward conditions. (E)-(F) Scatterplots of the baseline activity of BS R-neurons against the delay activity in the preferred (E) and non-preferred reward conditions. (G)-(H) The activity of NS R-neurons in the late cue period against the baseline activity in the preferred (G) and non-preferred (H) reward conditions. (I)-(J) The activity of NS R-neurons in the delay period against the baseline activity in the preferred (I) and non-preferred (J) reward conditions. Filled circles and triangles indicate statistical significance (sig., Mann Whitney *U* test, P < 0.05) and open ones indicate no statistical significance (n.s., P > 0.05).

R-neurons encoded reward information regardless of the visual properties of the stimulus. In our database, about half of the R-neurons showed higher activity in large reward trials, and the other half had higher activity in small reward trials. Fig 4A shows population histograms of 59 BS neurons (red curves) and of 18 NS neurons (blue curves) that were identified as R-neurons in the cue period. Fig 4B shows population histograms of 43 BS neurons and of 19 NS neurons that were identified as R-neurons in the delay period but not in the cue period. Both BS and NS R-neurons discriminated the preferred from non-preferred reward conditions in the cue (mean normalized activity for BS: 0.50 (preferred), 0.21 (non-preferred), Mann Whitney U test, P < 0.001; for NS: 0.57 (preferred), 0.3 (non-preferred), Mann Whitney U test, P < 0.001) and delay periods (mean normalized activity for BS: 0.47 (preferred), 0.16 (non-preferred), Mann Whitney U test, P < 0.001; for NS: 0.54 (preferred) and 0.22 (non-preferred), Mann Whitney U test, P < 0.001).

#### BS R-neurons encode the preferred reward information

Next we examined what type of reward information BS R-neurons encoded. To investigate this question, we compared the pre-cue activity (-300-0ms prior to the first cue onset) with the post-cue activity on the same trials (see Materials and methods). From the population level, these 59 BS R-neurons significantly increased their firing rates in the late cue period (300-500ms from the first cue onset) compared to firing rates in the fixation period under the preferred reward condition (see the solid red curve in Fig 4A, median baseline rate: 5.5 Hz, median discharge rate in the late cue period: 12.4 Hz, Wilcoxon signed rank test, *P* < 0.001), but showed no activity changes under the non-preferred reward condition (see the dashed red curve in Fig 4A, median baseline rate: 4.8 Hz, median discharge rate in the late cue period: 4.1 Hz, Wilcoxon signed rank test, P = 0.874). The activity of individual neurons in the fixation and late cue periods was shown in the preferred (Fig 4C) and non-preferred (Fig 4D) reward conditions, respectively. Consistently, about 80% of BS R-neurons (47/59) showed significantly increased activity in the late cue period relative to the baseline activity (Mann Whitney U test, P < 0.05) in the preferred reward condition (see filled circles above the diagonal line in Fig 4C). In contrast, the majority of these neurons (72.3%, 45/59) had no significant activity changes between the two periods in the non-preferred reward condition (see open circles in Fig 4D, P > 0.05). The incidence of significant neurons was higher in the preferred than in non-preferred reward conditions (preferred: 79.7%, non-preferred: 23.7%, χ^2^-test, P < 0.01). The proportion of insignificant neurons was lower in the preferred reward condition (preferred: 15.5%, non-preferred: 79.7%, χ^2^-test, P < 0.01).

The BS R-neurons identified in the delay period had the same response pattern as did those BS R-neurons in the cue period. We observed the population activity of these 72 BS R-neurons were significantly higher in the delay period than in the fixation period under the preferred reward condition (baseline: 4.8 Hz, the delay period: 11.3 Hz, Wilcoxon signed rank test, *P* < 0.001), and the activity in the two periods did not significantly differ in the non-preferred reward condition (baseline: 4.2 Hz, the delay period: 3.4 Hz, Wilcoxon signed rank test, P = 0.189). In the preferred reward condition, 82% of individual neurons (59/72) had significantly greater activity in the delay period than in the fixation period (see filled triangles above the diagonal line in Fig 4E, Mann Whitney U test, P < 0.05). In the non-preferred reward condition, however, over 70% of them (52/72) did not show activity differences in the two periods (see open triangles in Fig 4F, P > 0.05). The incidence of significant neurons was higher and the incidence of insignificant neurons was lower in the preferred reward condition (χ^2^-test, P < 0.01). The activity patterns of population and individual BS R-neurons consistently suggested that the majority of BS R-neurons represented the preferred but not the non-preferred reward information in the late cue and delay periods.

#### NS R-neurons encode the non-preferred reward information

The NS R-neurons displayed a response pattern that differed from the response pattern of the BS R-neurons. In the non-preferred reward condition, the population activity of the NS R-neurons in the baseline period was significantly higher than the activity in both the late cue and delay periods (see dashed blue curves in Fig 4A and 4B, 18 NS R-neurons in the cue period: median baseline rate: 27.2 Hz, median rate in the late cue period: 16.6 Hz, *P* < 0.01; 30 NS R-neurons in the delay period: baseline: 24.9 Hz, the delay period: 10.3 Hz, *P* < 0.001, Wilcoxon signed rank test). In the preferred reward condition, the baseline activity of the NS R-neurons was not significantly different from the activity either in the late cue period (see the solid blue curve in Fig 4A, baseline: 23.2; the late cue period: 32.9 Hz, Wilcoxon signed rank test, P = 0.094) or in the delay period (see the solid blue curve in Fig 4B, baseline: 23.4 Hz, the delay period: 23.9 Hz, Wilcoxon signed rank test, *P* = 0.271). For individual neurons, the majority of the NS R-neurons showed significantly higher baseline activity relative to the activity in the late cue period (see filled circles under the diagonal line in Fig 4H, 77.8%, 14/18, Mann Whitney U test, P < 0.05) or in the delay period (see filled triangles under the diagonal line in Fig 4J, 86.7%, 26/30, P < 0.05) under the non-preferred reward condition. In contrast, under the preferred reward condition, the baseline activity of the majority of the same population neurons was not significantly different from the activity either in late cue period (see open circles in Fig 4G, 72.2%, 13/18, Mann Whitney U test, P > 0.05) or in the delay period (see open triangles in Fig 4I, 70%, 21/30, P > 0.05). In the both late cue and delay periods, the incidences of significant neurons were lower and the incidences of insignificant neurons were higher in the preferred reward condition relative to that in the non-preferred reward condition (χ^2^-test, P < 0.01). The response patterns of NS R-neurons suggested that the majority of NS R-neurons represented the non-preferred but not the preferred reward information in the late cue and delay periods.

### BS and NS S-neurons

**Fig 5:**
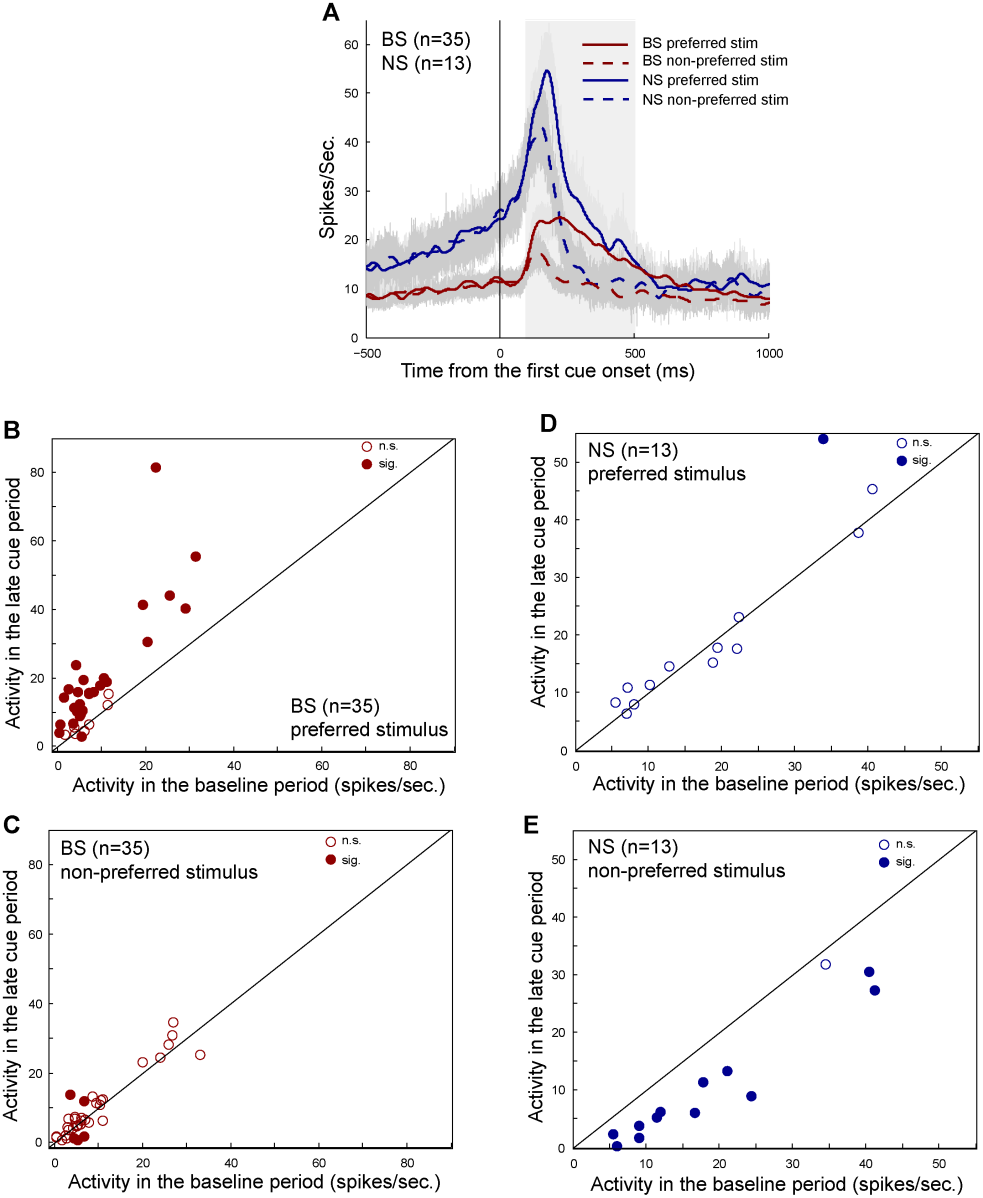
Response properties of BS and NS S-neurons in the cue period. (A) Population histograms of BS (red curves) and NS (blue curves) neurons that were identified as the stimulus type in the cue period. The neuronal activity was sorted by the preferred stimulus (solid curves) and the non-preferred stimulus (dashed curves). The shaded areas around the curves indicate SEM. The gray area indicates the cue period. (B)-(C) Scatterplots of the baseline activity of each BS S-neurons against the late cue activity to the preferred stimulus (B) and to the non-preferred stimulus (C). (D)-(E) Scatterplots of the activity of NS S-neurons to the preferred stimulus (D) and to the non-preferred stimulus (E). Filled circles indicate statistical significance (sig., Mann Whitney *U* test, P < 0.05) and open ones indicate no statistical significance (n.g., P > 0.05).

We found 35 BS and 13 NS S-neurons in the cue period, and 17 BS and 2 NS S-neurons in the delay period. These S-neurons showed stimulus selective activity regardless of the reward condition. Here we mainly focused on analyzing the neuronal activity in the cue period. Fig 5A shows the population histograms of 35 BS S-neurons and of 13 NS S-neurons. The averaged activity of both BS and NS S-neurons in the cue period discriminated the preferred from the non-preferred stimuli (BS mean normalized activity: 0.52 (preferred) and 0.26 (non-preferred), Mann Whitney U test, P < 0.01; NS mean normalized activity: 0.59 (preferred) and 0.31 (non-preferred), Mann Whitney U test, P < 0.01).

For these S-neurons, we also compared their baseline activity in the fixation period with their activity in the late cue period to demonstrate what type of stimulus information they encoded. In the late cue period, the population activity of BS neurons to the preferred stimulus was significantly higher than the baseline activity (see the solid red curve in Fig 5A, median baseline activity: 5.6 Hz, median firing rate in the late cue period: 14.1 Hz, Wilcoxon signed rank test, P < 0.01), while their activity to the non-preferred stimulus was at the baseline level (see the dashed red curve in Fig 5A, baseline: 6.1Hz, the late cue period: 6.6 Hz, Wilcoxon signed rank test, P = 0.7511). The NS S-neurons showed a different response pattern. Their population activity in the late cue period significantly decreased relative to the baseline activity when the non-preferred stimulus was presented as the first cue (see the dashed blue curve in Fig 5A, baseline: 16.7Hz, the late cue period: 6.2 Hz, Wilcoxon signed rank test, P = 0.0455), but was not different from the baseline activity when the preferred stimulus was presented (see the solid blue curve in Fig 5A, baseline: 18.8Hz, the late cue period: 15.2 Hz, Wilcoxon signed rank test, P = 0.874). The population activity suggested that BS S-neurons represented information of the preferred stimulus, while NS S-neurons encoded information of the non-preferred stimulus.

To determine whether individual neurons in the BS and NS groups consistently encode the same aspect of stimulus information (preferred or non-preferred), we compared the activity of each neuron in the fixation period with the activity in the late cue period. It was found that more than 70% (27/35) of BS S-neurons increased their responses to the preferred stimulus relative to the baseline activity (see filled circles above the diagonal line in Fig 5B, Mann Whitney U test, P < 0.05), while only 20% (7/35) of them did not change their activity in the two periods. In contrast, the majority of the same population neurons (85.7%, 30/35) did not show activity changes between the baseline period and the late cue period when the non-preferred stimulus was presented as the first cue (see open circles in Fig 5C), and only 14.3% (5/35) of them had significantly differential activity in the two periods. The incidence of significant neurons was higher and the incidence of insignificant neurons was lower in the preferred stimulus condition than in the non-preferred stimulus condition (χ^2^-test, P < 0.01). For the NS S-neurons, the most of them (92.3%, 12/13) fired at the baseline level in the late cue period when the preferred stimulus was presented as the first cue (see open circles in Fig 5D), while only one neuron increased its activity. In contrast, 12 out of 13 NS S-neurons (92.3%) decreased their activity in the late cue period when the non-preferred stimulus was presented (see filled circles under the diagonal line in Fig 5E), and only one neuron did not change its activity. The incidence of significant neurons was lower and the incidence of insignificant neurons was higher in the preferred stimulus than in the non-preferred stimulus (χ^2^-test, P < 0.01). These results indicated that BS and NS S-neurons encoded the preferred and non-preferred stimuli, respectively.

We further examined response properties of the 17 BS S-neurons identified in the delay period, but did not compare their activity to those of the NS S- neurons (only 2). During the delay period, firing rates of the BS S-neurons to the preferred stimulus significantly increased relative to the baseline firing rates (baseline: 6.95Hz, the delay period: 10.93Hz, Wilcoxon signed rank test, P < 0.01), and the firing rates to the non-preferred stimulus had no significant differences from the baseline level (baseline: 6.89Hz, the delay period: 6.33Hz, Wilcoxon signed rank test, P = 0.084). For these individual neurons, 76.5% (13/17) of them showed significantly higher activity in the delay period than in the fixation period when the preferred stimulus was presented as the first cue. When the non-preferred stimulus was shown as the first cue, over 80% (14/17) of them fired at the baseline level in the delay period. The response pattern of the BS S-neurons in the delay period was consistent with the response pattern of the BS S-neuron in the cue period. The results from the activity of population and individual neurons demonstrated that the majority of BS and NS S-neurons represented different stimuli (preferred vs non-preferred), the former increased their firing rates to represent the preferred stimulus, while the latter decreased their firing rates to represent the non-preferred stimulus.

### BS and NS SR-neurons

**Fig 6:**
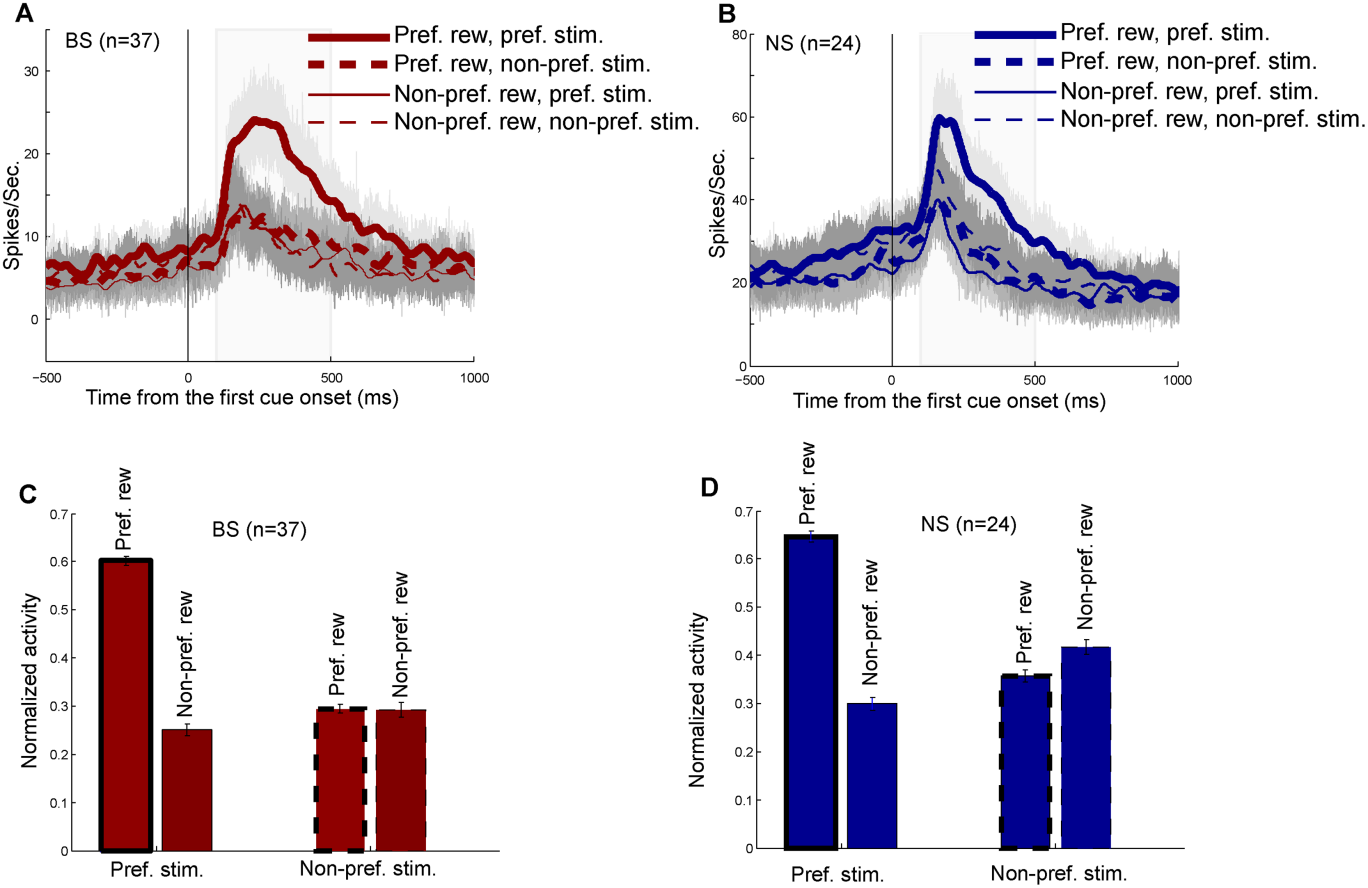
Response properties of BS and NS SR-neurons in the cue period. (A) Population histograms of BS SR-neurons sorted by four conditions: the preferred reward with preferred stimulus (thick solid curve), the preferred reward with non-preferred stimulus (thick dashed curve), the non-preferred reward with preferred stimulus (thin solid curve) and the non-preferred reward with non-preferred stimulus (thin dashed curve). (B) Population histograms of NS SR-neurons. The activity was sorted with the same four conditions as in (A). (C) Averaged activities of BS SR-neurons in the cue period. Four bars indicate the activities in the four conditions. (D) Average activities of NS SR-neurons in the cue period.

There were 37 BS and 24 NS SR-neurons in the cue period. The SR-neurons encoded both stimulus and reward information simultaneously. For each neuron, we defined its preferred reward and stimulus. The preferred reward was referred to as the reward condition that elicited the higher firing rate by the stimuli (A1 and A2), and the preferred stimulus was defined as the stimulus that evoked greater activity in the preferred reward condition. Here we focused on analyzing the activity of the SR-neurons identified in the cue period. Population histograms of BS and NS SR-neurons are shown in Fig 6A and 6B, respectively. Both the BS and NS neurons showed the highest activity in the preferred stimulus and reward conditions. Fig 6C and 6D show the averaged activity of BS and NS neurons in the cue period. Results of two-way ANOVA (reward x stimulus) demonstrated that neuronal activity seen in Fig 6C and 6D had significant main effects of stimulus and reward, and a significant interaction between the two factors (P < 0.01). Post-hoc tests with Bonferrini correction indicated that both BS and NS SR-neurons showed significantly higher activity in the preferred than in non-preferred reward conditions when the preferred stimulus was presented as the first cue (P < 0.01). When the non-preferred stimulus was presented, neither BS nor NS neurons had significantly different activity between the two reward conditions (P > 0.05). The results suggested that BS and NS neurons encoded reward information for the preferred stimulus, but not for the non-preferred stimulus.

### Discrimination index and latency of BS and NS neurons

**Fig 7:**
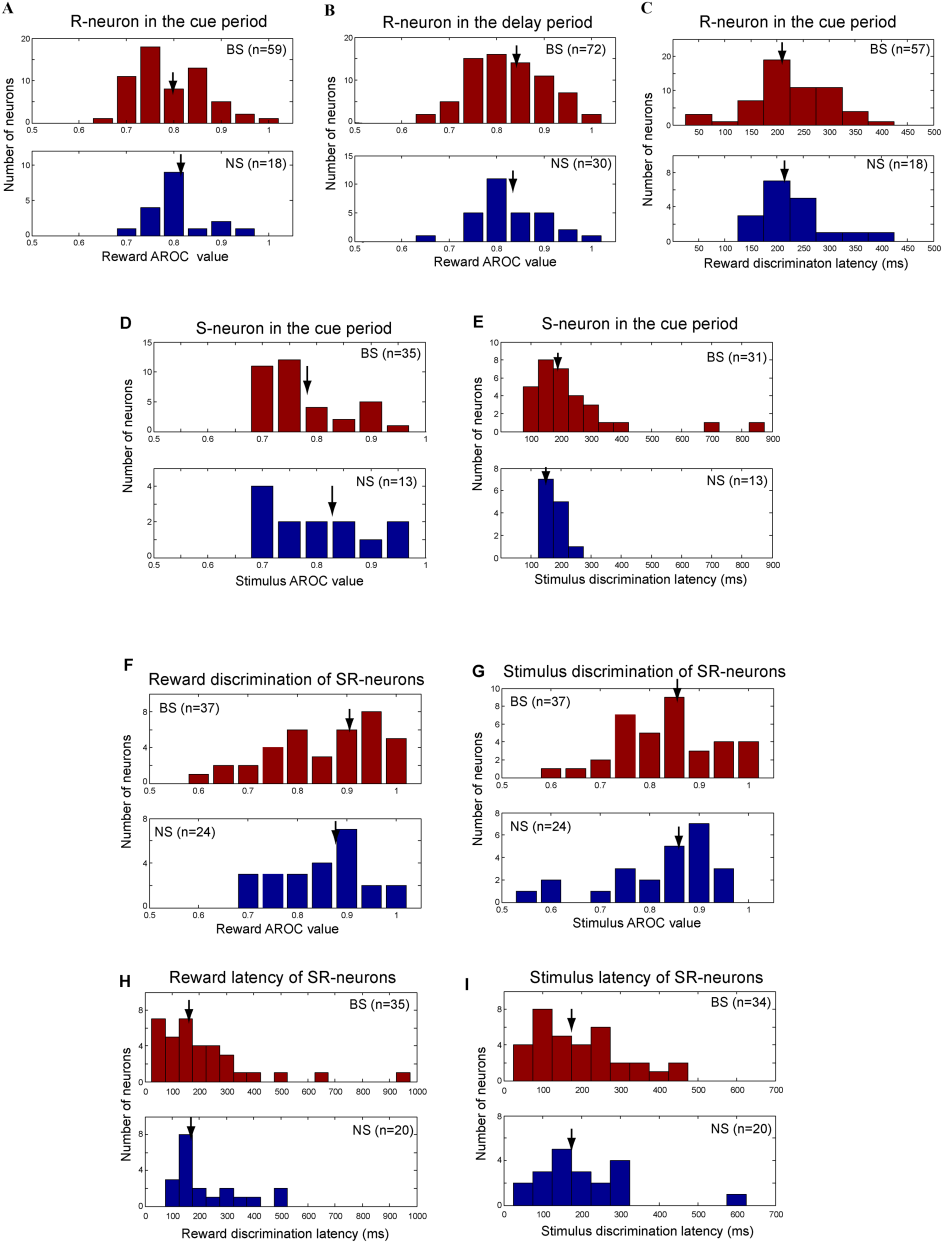
Distributions of AROC values and discrimination latencies for BS and NS neurons. (A) Distributions of reward AROC values of BS (upper panel) and NS (bottom panel) R-neurons during the cue period. (B) Distributions of reward AROC values of BS (upper panel) and NS (bottom panel) R-neurons during the delay period. Reward AROC values are not significantly different between BS and NS R-neurons either in the cue period or in the delay period. (C) Distributions of reward discrimination latencies of BS (upper panel) and NS (bottom panel) R-neurons that were identified in the cue period. Two BS neurons were excluded for the latency count. Reward discrimination latencies did not significantly differ between the two classes of neurons. (D) Distributions of stimulus AROC values of BS (upper panel) and NS (bottom panel) S-neurons. Stimulus AROC values were no significant differences between the two classes. (E) Distributions of stimulus discrimination latencies of BS (upper panel) and NS (bottom panel) S-neurons. The arrow indicates the median value in each distribution. (F) Distributions of reward AROC values of BS (upper panel) and NS (bottom panel) SR-neurons. (G) Distributions of stimulus AROC values of BS (upper panel) and NS (bottom panel) SR-neurons. (H) Reward discrimination latencies of BS (upper panel) and NS (bottom panel) SR-neurons. (I) Stimulus discrimination latencies of BS (upper panel) and NS (bottom panel) SR-neurons. The arrow in each distribution indicates the median value. Totally 35 BS and 20 NS neurons were recruited for reward latency count. And 34 BS and 20 NS neurons were recruited for stimulus latency count.

Both the BS and NS R-neurons showed reward-modulated activity. To quantify reward discrimination ability of each R-neuron, an AROC value was calculated in the cue (Fig 7A) and delay periods (Fig 7B) by the ROC method for each BS and NS R-neurons (see Materials and methods), respectively. The AROC values of BS R-neurons did not differ significantly from the AROC values of NS R-neurons either in the cue period (BS median AROC value: 0.759, NS median AROC value: 0.815, P = 0.6174, Mann Whitney U test) or in the delay period (BS: 0.841, NS: 0.835, P = 0.9736, Mann Whitney U test). The ROC analysis reveals the trial-by-trial variability of discrimination between the two reward conditions. The results indicated that both BS and NS R-neurons had similar reliability to distinguish the preferred from non-preferred reward conditions across trials. We also calculated the reward discrimination latencies for the two classes of neurons. The latencies were not significantly different between the two groups (Fig 7C, the median latency of BS neurons: 210ms, the median latency of NS neurons: 215ms, Mann Whitney *U* test, P = 0.8133).

Fig 7D shows the AROC values between the preferred and non-preferred stimuli for BS (upper panel) and NS S-neurons (bottom panel). The distributions of AROC values in the cue period did not significantly differ between the two classes of neurons (BS median AROC value: 0.782, NS: 0.829, P = 0.2965, Mann Whitney *U* test), indicating that the both BS and NS S-neurons had equal ability to discriminate the preferred stimulus from the non-preferred stimulus. This result was consistent with previous reports that both LPFC BS and NS neurons were equally likely to reliably represent motion direction [11,12]. The stimulus discrimination latencies in two classes of neurons had no significant difference either (Fig 7E, Mann Whitney *U* test, P = 0.2672).

We calculated AROC values between the preferred and non-preferred stimuli under the preferred reward condition and AROC values between the preferred and non-preferred reward under the preferred stimulus for both classes of SR-neurons, respectively. In the cue period, the AROC values of BS SR-neurons did not significantly differ from the AROC values of NS SR-neurons either between the two reward conditions (Fig 7F, Mann Whitney *U* test, P = 0.5156) or between the preferred and non-preferred stimuli (Fig 7G, Mann Whitney *U* test, P = 0.9882). Neither reward discrimination latencies (Fig 7H) nor stimulus discrimination latencies (Fig 7I) were significantly different between the two classes of SR-neurons (Mann Whitney *U* test, P > 0.3). These results demonstrated that both BS and NS SR-neurons had equal reliability to encode stimulus and reward information.

### Relation of reward-related activity to saccadic metrics

The four monkeys consistently showed significantly higher correct rates of the first choice in large than in small reward trials (see Fig 1). Response times (the duration from the fixation offset to the monkey’s eyes saccade to the target) and saccadic peak velocities in the first choice were modulated by reward amount for each monkey. The monkey Hop showed shorter response times and faster peak velocities in large than in small reward trials; the monkey Tap showed longer response times and slower peak velocities in large reward trials; for the monkey Tom, there were no significant response time differences in the two reward trials, but slower peak velocities in large reward trials; and the monkey Zep had longer response times and faster peak velocities in large reward trials. One interesting question is that for each neuron whether its reward-related activity in the cue period or in the delay period was correlated with saccadic parameters (e.g., saccadic response time, saccadic peak velocity and direction) or not. To investigate this issue we performed a multiple linear regression method with two models (see Materials and methods): 1) firing rate as a function of stimulus and reward size, and 2) firing rate incorporating four variables: left saccadic response time, right saccadic response, left peak velocity and right peak velocity. We found that all of NS R-neurons and 99.2% of BS R-neurons showed a significant goodness of fit with Model 1 (F-test, P < 0.05), and only 2.08% of NS R-neurons and 3.81% of BS R-neurons had a significant goodness of fit with Model 2 (F-test, P < 0.05). For those SR neurons, the activity of 62.3% of BS and 46.6% of NS was significantly fitted with Model 1, respectively. And None of BS SR-neurons fitted well with Model2, and 10% of NS SR-neurons fitted significantly with Model 2. The results indicated that the activity of the two types of cells was not correlated with motor kinematics, but with the stimulus and the reward amount, consistent with the results in previous reports [22,24].

From the behavioral level, the monkeys correctly predicted reward values of the first cues (A1 and A2) from the first SPATs after reward instruction of C1 and C2 in current block regardless of the stimulus-reward contingency in the previous block, showing higher correct rates of the first choice in large than in small reward trials (see Fig 2 in Pan et al. [25]). Correspondingly, it was found that both the BS and NS R-neurons were able to distinguish one reward condition from the other from the first SPATs after reward instruction of C1 and C2 in one block, consistent with reward modulated behavioral changes. The match between neural activity and behavioral changes suggested that both BS and NS cells had contributions to the monkey’s reward predictive behavior.

## Discussion

In this study, single-unit activity was recorded in the LPFCs of the monkeys performing the reward prediction task. Based on their extracellularly recorded spike waveforms, LPFC neurons were classified separately into BS and NS groups. The observed physiological differences between the two groups of neurons suggested that the sample of BS cells mostly contained pyramidal cells, while the sample of NS cells mostly contained interneurons. Both the BS and NS neurons had similar incidences of the following three types of neurons: R-neurons, S-neurons and SR-neurons. The BS and NS neurons had equal reliability (similar AROC values) to distinguished one reward condition from the other reward condition, one stimulus from the other, indicating that both of them were involved in reward and stimulus processing in the LPFC. The BS R-neurons raised their firing rates to represent the preferred reward information, while the NS reward cells reduced their discharge rates to represent the non-preferred reward information. Similarly, the BS S-neurons encoded the preferred stimulus by increasing their firing rates and the NS S-neurons encode the non-preferred stimulus by decreasing their firing rates relative to the baseline activity. The results suggested that BS and NS cells encode the preferred and non-preferred information via distinct mechanisms in the task.

### Physiological differences between BS and NS cells

LPFC neurons were objectively classified into the BS and NS groups by a linear classifier (k-means, k = 2) based on their shapes of averaged spike waveforms. The observed differences in physiological responses between the two classes of neurons indicate that they resembled putative pyramidal cells and interneurons. First, the distributions of waveform durations were bimodal (see Fig 2F–2I). The NS neurons had shorter durations, and the BS neurons had longer durations. Intracellular and whole-cell recording studies with histological methods have demonstrated that pyramidal cells have broader waveform durations than do interneurons [2,4,36–38]. Therefore, most of the BS cells in current study should be pyramidal cells and most of NS cells should be interneurons. Second, the NS neurons showed significantly higher firing rates (see Fig 3A–3C), which is a typical characteristic of interneurons [36,37,39]. Intracellular studies have found that interneurons have higher firing rates than pyramidal cells when stimulated by current injection or visual stimuli [40]. The mean baseline activity of BS neurons (5.4 Hz) was also in line with the typical mean rate of 4-8Hz for pyramidal cells [41,42]. Third, intracellular and anatomical studies have estimated that roughly 70–80% of cortical neurons are pyramidal cells and that the remaining neurons are interneurons [36,43–45]. Consistent with these findings, we found that 77.7% of our sample neurons were BS cells, and 22.3% of them were NS cells. Fourth, the NS neurons showed shorter visually evoked latencies than did the BS neurons. Intracellular experiments have found that interneurons have significantly larger and faster EPSPs than pyramidal cells [32]. This may cause a lower threshold for spike generation and shorter latencies for EPSP-spike coupling in interneurons. Fifth, the BS cells had significantly higher local variation values than did the NS cells, indicating that the BS cells showed a relatively burst-like pattern [30]. In addition, the BS cells showed longer peak mode time in ISI distributions than did the NS cells. These spike train statistical differences between the two classes of neurons were in agreement with statistical observations in pyramidal cells and interneurons [31,32,46]. Overall, the distinct properties in spike waveforms, response patterns and spike trains between the two classes of neurons suggested that our sample of LPFC neurons were classified into putative pyramidal cells and putative interneurons correctly based on their spike waveforms.

### Reward information encoded by BS and NS cells

Both the BS and NS R-neurons discriminated the preferred from non-preferred reward conditions (see Fig 4A and 4B). Their population activities were stronger in the preferred than non-preferred reward conditions. To determine whether a neuron encodes reward information or not, we usually compare the neuronal activity on trials containing different rewards. If one neuron shows activity differences on different rewarded trials, we say this neuron encodes reward information; otherwise, this neuron does not encode reward information. This type of analysis has been often used in the literature of reward experiments [23–25], but it does not tell us what type of reward information the R-neuron encodes. To further investigate this issue, the activity of an R-neuron is compared in different time epochs on the same trials.

We compared the activity of BS and NS neurons in the fixation period with the activity in the late cue period and with the activity in the delay period. The neuronal activity at the fixation period did not reflect any reward information. After the first cue presentation, the monkeys were able to predict the reward amount in current trial, and therefore the neuronal activity could encode reward information during the cue and delay periods. An activity change from the fixation period to the cue period or to the delay period would indicate the reward condition in current trial. If there is no such change, the neuron does not predict the reward amount that the monkeys would receive at the end of the trial. Interestingly, the BS R-neurons increased their discharge rates in the late cue and delay periods relative to the baseline rates in the preferred reward condition, while they did not change their activity in the non-preferred reward condition (see Fig 4C, 4D, 4G and 4H). This pattern of results indicated that the activity of BS R-neurons encoded the preferred reward information but not the non-preferred reward information. On the contrary, in the non-preferred reward condition the NS R-neurons decreased their firing rates relative to the baseline rates, but they did not change their activity in the preferred reward condition (see Fig 4E, 4F, 4I and 4J). This pattern of results suggested that the NS R-neurons encoded the non-preferred reward information, but not the preferred reward information. Our findings indicated that the BS and NS R-neurons complementarily encoded the reward information, with each type of neuron representing reward information via different mechanisms.

### Stimulus information encoded by BS and NS cells

The BS and NS S-neurons represented stimulus information via a mechanism similar to that which the R-neurons used to encode reward information. When the activities were compared on trials containing the preferred and non-preferred stimuli, both the BS and NS S-neurons showed higher activity to the preferred stimulus. When we compared the baseline activity in the fixation period with the activity in the late cue period, we found that the BS S-neurons increased their activity to the preferred stimulus but maintained the baseline level response to the non-preferred stimulus (see Fig 5B and 5C). In contrast, the NS S-neurons decreased their activity in the late cue period to the non-preferred stimulus but maintained baseline activity to the preferred stimulus (see Fig 5D and 5E). Although the number of NS S-neurons was small (n = 13) in the cue period, response pattern differences between the two classes of neurons reached statistical significance. Moreover, the BS S-neurons identified in the cue and delay periods showed consistent response properties to the preferred and non-preferred stimuli. These results together suggested that the BS S-neurons only encoded the information of the preferred stimulus and that the NS stimulus neurons only represented the information of the non-preferred stimulus.

Some studies that examined responses of both types of cells to preferred and non-preferred visual stimuli reported more complex response patterns of BS and NS neurons. In a motion direction discrimination task [11], both BS and NS neurons in the LPFC increased their firing rates to the preferred and anti-preferred direction motions relative to the baseline activity in the fixation period. There was no suppressive population activity to the anti-preferred direction motion in NS cells. In another numerical categorization task [10], LPFC BS and NS neurons could encode various numerosities by their graded activity. Responses of both types of cells to the least-preferred numerosity were depressed compared to the baseline activity. Those observations were inconsistent with our findings that both BS and NS utilized a binary strategy to encode the preferred and non-preferred information for both reward and stimulus. One possible explanation was that the monkey had to memorize and discriminate several visual stimuli simultaneously in those tasks [10,11], which enabled LPFC BS and NS neurons to represent visual information in the complex way. In our task, there were two visual stimuli (A1 and A2) and two reward conditions (large and small reward). BS and NS neurons were able to represent stimulus and reward information via this binary method. This type of binary coding strategy is consistent with the task structure.

### Functional specialization of BS and NS neurons in the reward prediction task

By the method of multiple regression analysis, we established that the reward-related activity of NS neurons or of the BS neurons in the cue period or in the delay period was not correlated with motor outputs such as saccadic response time, directions and peak velocities [22,24]. The activity change between the two reward conditions could not be explained by variation in those parameters, but explained by the type of visual stimulus and its associated reward amount, although response time and peak velocities were different between large and small reward trials. These results indicated that both the BS and NS cells encoded reward information associated with each visual stimulus, and was not directly correlated with motor kinematics. The both cell classes discriminated the two reward conditions from the first SPATs after reward instruction of C1 and C2 in one block, consistent with the monkeys’ reward-prediction behavioral patterns. Furthermore, either the stimulus or reward discrimination ability was not significantly different between the two cell groups. Their discrimination latencies were equal between the two cell classes (see Fig 7), indicating that the NS cells had critical contributions to the processing of stimulus and reward information in the PFC, as well as did the BS cells. The NS cells had high baseline firing rates and represented the non-preferred reward information by reducing their activity, while the BS cells having low baseline firing rates increased their activity to represent the preferred reward information. This type of encoding strategy is less energy consumption and more stable to represent stimulus and reward information in working memory. It was more efficient to compare with other strategies that both BS and NS neurons could increase their activity to represent the preferred and non-preferred reward information.

The properties of NS cells and their interactions with BS cells in the PFC have been studies in monkeys performing various cognitive tasks [10,15]. These studies showed that both the cell classes were able to carry cognitive signals (e.g. visual stimulus or spatial information) and demonstrated the importance of inhibition from NS cells to BS neurons to shape the property selectivity of BS cells in the PFC [10,14]. Two types of inhibitory microcircuits, known as “feedforward” and “feedback” inhibition, have been found in primate prefrontal cortex [47] and mouse prefrontal cortex [48]. These circuits consist of excitatory pyramidal cells and inhibitory interneurons. It has been hypothesized that the feedback inhibition is involved in controlling local excitability and the global balance within a network. The feedforward inhibition is thought to narrow the temporal window for action potential initiation in pyramidal cells and sharpen their outputs [41,49]. Both interneurons and pyramidal cells may simultaneously receive excitatory inputs, but that interneurons may respond earlier due to their faster channel kinetics. Therefore, interneurons may be able to inhibit or even prevent pyramidal cell firing. This inhibition from interneurons may then suppress the firing of pyramidal cells to non-optimal excitatory inputs but the most optimal one, which may improve the selectivity of pyramidal cells [50]. While our data did not provide insights into whether the response patterns emerged independently in the two cell classes or were a product of interactions between them, it is likely that the NS and BS cells recorded in this reward prediction task share a common cellular mechanism found in previous studies [10,14] that interneurons could regulate outputs of pyramidal cells.

In summary, LPFC neurons were classified as either BS or NS cells based on their extracellularly recorded spike waveforms. The encoded information related to stimulus and reward was similar between the BS and NS classes in term of proportion of neurons. The two classes of neurons had equal reliability in discrimination between the preferred and non-preferred reward and stimulus information. From activity at both the population and at the individual cell level, BS and NS neurons represented different aspects of reward and stimulus information using distinct mechanisms. BS neurons increased their activity relative to baseline to encode the preferred information, whereas NS neurons decreased their activity to represent the non-preferred information. The response properties of BS and NS neurons and their interactions could have important implication for meditating the balance between excitation and inhibition in the prefrontal microcircuits that ultimately shape cognitive functions in the PFC [50–52]. In this study, we had not enough neuron pairs (i.e. BS-BS, BS-NS, NS-NS) recorded simultaneously to investigate interactive functions among BS and NS neurons in a circuit involved in stimulus and reward processing. Further study is needed to clarify the neuronal mechanism how BS and NS neurons connect each other in a microcircuit to represent stimulus and reward information using the complementary strategy.
